# EVEREST: automatic identification and classification of protein domains in all protein sequences

**DOI:** 10.1186/1471-2105-7-277

**Published:** 2006-06-02

**Authors:** Elon Portugaly, Amir Harel, Nathan Linial, Michal Linial

**Affiliations:** 1 School of Computer Science & Engineering, The Hebrew University of Jerusalem, Jerusalem, Israel; 2 Department of Biological Chemistry, Institute of Life Sciences, The Hebrew University of Jerusalem, Jerusalem, Israel

## Abstract

**Background:**

Proteins are comprised of one or several building blocks, known as domains. Such domains can be classified into families according to their evolutionary origin. Whereas sequencing technologies have advanced immensely in recent years, there are no matching computational methodologies for large-scale determination of protein domains and their boundaries. We provide and rigorously evaluate a novel set of domain families that is automatically generated from sequence data. Our domain family identification process, called EVEREST (EVolutionary Ensembles of REcurrent SegmenTs), begins by constructing a library of protein segments that emerge in an all vs. all pairwise sequence comparison. It then proceeds to cluster these segments into putative domain families. The selection of the best putative families is done using machine learning techniques. A statistical model is then created for each of the chosen families. This procedure is then iterated: the aforementioned statistical models are used to scan all protein sequences, to recreate a library of segments and to cluster them again.

**Results:**

Processing the Swiss-Prot section of the UniProt Knoledgebase, release 7.2, EVEREST defines 20,230 domains, covering 85% of the amino acids of the Swiss-Prot database. EVEREST annotates 11,852 proteins (6% of the database) that are not annotated by Pfam A. In addition, in 43,086 proteins (20% of the database), EVEREST annotates a part of the protein that is not annotated by Pfam A. Performance tests show that EVEREST recovers 56% of Pfam A families and 63% of SCOP families with high accuracy, and suggests previously unknown domain families with at least 51% fidelity. EVEREST domains are often a combination of domains as defined by Pfam or SCOP and are frequently sub-domains of such domains.

**Conclusion:**

The EVEREST process and its output domain families provide an exhaustive and validated view of the protein domain world that is automatically generated from sequence data. The EVEREST library of domain families, accessible for browsing and download at [1], provides a complementary view to that provided by other existing libraries. Furthermore, since it is automatic, the EVEREST process is scalable and we will run it in the future on larger databases as well. The EVEREST source files are available for download from the EVEREST web site.

## Background

The study of proteins and their properties is of uttermost importance for biology, and computational tools have become an important ingredient in this endeavor. A very large number of protein sequences are already known: About 200,000 at the highly curated, non-redundant, Swiss-Prot section of the UniProt Knowledgebase (UniProtKB) release 7.2 [2], and an order of magnitude more at the genomic-based, non-curated TrEMBL section of UniProtKB. However, our knowledge of higher properties of proteins, such as their 3D structure and function is much more fragmentary. Thus the number of UniProtKB release 7.2 proteins whose structure is known is only about 11,000 (of those, about 9,000 are from the Swiss-Prot section). It is, of course, much harder to experimentally derive such information. Needless to say, we are still far from being able to deduce a protein's structure or function from its sequence. It is hard to overstate the impact that such methods would have on the field, since the vast amount of protein sequence data would immediately translate into a much more profound biological comprehension of proteins and their functionalities.

Our approach to the problem of deducing structure/function from sequence is based on inference by homology. The basic idea is to infer a protein's higher properties from those of other proteins which have similar sequences. However, current sequence comparison techniques are limited in their range and applicability. For many proteins such techniques can find no similar protein from which to infer information about structure or function. Only 55% of the amino acid positions in the Swiss-Prot segment of UniProtKB (release 7.2) can be aligned by BLAST [3] to any sequence with known structure, at a threshold of E-score below 0.1 (sequences of know structure obtained from PDB [4] on Feb. 2006). Note that this is a relaxed level of statistical confidence and is certain to introduce a large number of false positives. Even Pfam A with all its powerful manually tuned search tools, leaves 38% of the amino acids in the Swiss-Prot database unannotated.

Additional complexity results from the fact that proteins are typically composed of several subunits, called domains. The literature in protein science teems with definitions that attempt to capture the correct notion of a protein domain. We later return to the issue of the "appropriate" definition of a domain. The computational problem of correctly dissecting a protein sequence into its domains is still largely open. It is of great importance since both the function and the structure of a protein can be inferred quite well from the function and structure of its constituent domains. Structural prediction algorithms may benefit from the definition of protein domains and sub-domains [5]. In the scope of the structural genomics initiatives, the identification and classification of domains from sequence is crucial for the selection of proper crystallography targets, and the definition of domain boundaries is essential for successful crystallization.

**Figure 1:**
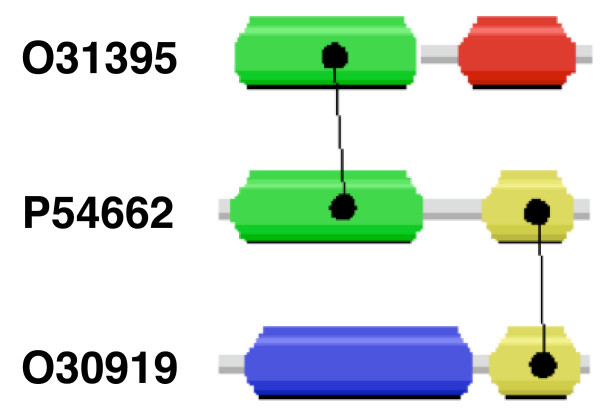
**Multi domain proteins and the trap of false transitivity**. Three bacterial proteins are shown. Boxes represent domains, colored by their Pfam classification. Red = *C terminal transcriptional regulatory domain*; Green = *Response regulator receiver domain*; Yellow = *luxR family*, Blue = *Autoinducer binding domain*; Thin lines mark similarity of domains. The protein [Swiss-Prot:P54662] is similar to each of the two other proteins via a different domain, therefore careless use of transitivity might lead to the false conclusion that [Swiss-Prot:O31395] is similar to [Swiss-Prot:O30919].

Dissecting protein sequences into their domains would also help avoid false transitivity in large-scale efforts of clustering and classifying protein sequences. The difficulty stems from the fact that various combinations of similar domains may appear in distinct proteins. Figure 1 shows an example of three proteins containing different combinations of four domain families. This evolutionary "mix and match" of domains yields new proteins that are comprised of existing molecular building blocks.

As already mentioned, many publications have considered protein domains. The various definitions of a protein domain suggested by different authors do not always coincide and are not always even precisely stated [6]. Since the only raw data we use for this project consists of protein sequences, our choice of definition is rather natural. Namely, for us a domain is a continuous sequence of amino acids that recurs (non trivially) in the protein space. Thus, our domains are evolutionary in nature – segments of protein that are conserved and reused throughout evolution. We later comment on the correlation between the present definition and those adopted by others. A major source of difficulty in discerning protein domains is their hierarchical nature. A domain often has several well-defined and recurring sub-domains. Also, several domains may consistently and repeatedly appear together in specific combinations. Likewise, domain families are also hierarchical, several families may together form a super-family, and these may combine to yet another level of classification.

Let us return to the limitations of the existing sequence comparison techniques. Transitivity of similarity among proteins can be used to enhance similarity detection – if proteins **A **and **B **are known to be similar, and proteins **B **and **C **are known to be similar, transitivity would imply that proteins **A **and **C **are similar. However this transitivity should be used with care, so as to avoid two pitfalls. In a false match two proteins are considered similar, though they are biologically unrelated. Careless application of transitivity can amplify the effect of false matches. We must also beware the "trap of false transitivity" that is due to the way proteins are comprised of several domains, as illustrated by Figure 1. Careless use of transitivity entails a similarity between [Swiss-Prot:O31395] and [Swiss-Prot:O30919], although they share no common domains.

### Previous work

Several systems that define protein domains and classify them exist. Databases such as Pfam A [7] and SMART [8] offer comprehensive collections of families that were compiled by human experts, with the aid of computational tools (see review in [6]). These methods provide high quality definitions that are most useful for biologists. However they incorporate a great deal of human labor and expertise and require external information to identify new domain families. We use Pfam A, as well as the structure based classification provided by SCOP [9] as gold standards for evaluating our performance as well as the performance other competing systems.

Against which automatic systems that define domains and classify them can EVEREST be compared? The obvious candidates are the pioneering DOMO [10], the ProDom algorithm [11] that was adopted by Pfam and forms Pfam B, and the more recent ADDA [12]. DOMO is inappropriate for this purpose, since it is no longer up-to-date, and has performed poorly on preliminary tests we have conducted. It would be natural to compare EVEREST's performance with that of ProDom and Pfam B. Unfortunately, there seems to be no systematic evaluation of the quality of ProDom and Pfam B against any comprehensive reference set in the literature. (The only exception we are familiar with is a relatively brief discussion in [13]). Furthermore, the ProDom and Pfam B databases are created with full knowledge of Pfam A and SCOP. Therefore, it is impossible to post-hoc evaluate them against Pfam A or SCOP. Consequently, there is no ground for comparison with EVEREST here. Under these circumstances, the only alternative system against which we can compare EVEREST is ADDA. This indeed is the yardstick we use.

Nagarajan and Yona [14] developed a neural-network based method to parse a protein sequence into its domains using heterogeneous sources of information. CHOP [15] aims for the same goal using sequence alignments to known sequences of varying quality. Both methods focus on the accurate determination of domain boundaries, and do not attempt to classify the domains. We undertake the reciprocal task and wish to accurately classify the domain. For our purposes domain boundaries need to be accurate enough only so as to not interfere with the domains' classification.

### Goal and rationale

The goal of our research is to identify and classify all protein domains. We have developed EVEREST (EVolutionary Ensembles of REcurrent SegmenTs), an automatic method that identifies patterns within a protein sequence database and produces a set of statistical models, each modeling a sequence pattern that recurs in the database.

Our method utilizes two types of input – a database of protein sequences (typically a comprehensive database of all known sequences), and a collection of known domain families. The latter is used as a training set with which to exemplify to the system the notion of a domain family, but not to derive the characteristics of specific families. The performance of our system is then tested by evaluating its predictions on other known domain families.

There are several good reasons to seek an automatic system to determine and classify protein domains. The most obvious reason is that today's semi-manual techniques will become impractical as more and more data pours in as new genome projects reach completion. Also, automated methods are less prone to biases than semi-manual ones. Semi-manual methods require a predetermined seed for each family they define. This severely limits their potential to extend the repertoire of protein families beyond the boundaries of known biology. Automated methods are independent of today's biological knowledge, and thus have the potential of expanding it.

### Methodologies and concepts

Following are the main ideas incorporated into the EVEREST procedure:

#### Careful transitivity 1

We avoid false transitivity (see Background) by breaking the protein sequences into putative domains early in the process, and applying transitivity to them rather than to the whole sequences. The putative domains are refined during the process.

#### Careful transitivity 2

To reduce the adverse effects of false matches, we employ an average linkage algorithm. This algorithm is much less susceptible to noise than either single linkage or full linkage algorithms, and was successfully employed for whole protein sequence clustering in ProtoNet [16].

#### Selecting good candidate families using machine learning techniques

A random set of known families is provided as an additional input to the system. Based on this additional input, and using a boosting regression tool [17], our system generates its notion of a domain family. This allows us to first create many putative domain families, and then weed out those that do not match this notion.

#### Statistical models

Profile Hidden Markov Models (HMM) [18], are used to characterize our families. These statistical models have proven extremely useful in identifying distant similarities between protein sequences [19], and have boosted our system's performance significantly.

#### Iterative refinement

We use an iterative procedure to refine our results. The process begins by creating a database of putative domains. These putative domains are then clustered into a large set of putative domain families. Out of this set EVEREST selects those families matching the learned notion of domain family. A statistical model (HMM) is created for each of those families. We then iterate by using the statistical models to recreate the putative domains database and repeat the procedure.

We use the iterations to both improve the quality of the suggested domain families and reduce their number.

#### Expert voting

The families defined by our profile HMMs often overlap, i.e. several HMMs provide different descriptions of the same family. We identify sets of such overlapping HMMs, and let each one of them evaluate each domain found by any HMM in the set. We then define a family by accepting only domains with a good average score. As with our iterative refinement, this process both improves the quality of our families, and reduces their number.

**Figure 2:**
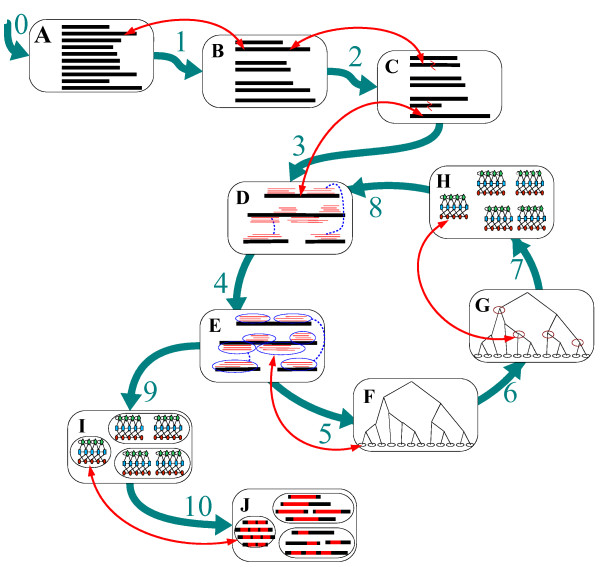
**The EVEREST process**. The turquoise arrows represent the steps of the procedure as detailed in the text. The panels represent the state of the data between the steps. Red arrows connect two manifestations of the same object. **0 **Input: a database of protein sequences. **1 **A non-redundant sequence database is created. **2 **Internal repeats are removed from sequences. **3 **Segments recurring in the database are identified using pairwise sequence comparison. **4 **Within each protein, segments are grouped by position into putative domains. **5 **Putative domains are clustered into candidate domain families. **6 **Machine learning is used to select the best of the candidate domain families. **7 **An HMM is built for each selected domain family. **8 **The input database is scanned by each HMM, recreating a segments database. The segments defined by each HMM are considered a domain family. **9 **Steps 4–8 are iterated three times. The domain families defined by the third iteration HMMs are clustered into sets of overlapping families. **10 **Final domain families are defined by a voting of all the HMMs of each set. See section **The EVEREST Process **for further details.

Figure 2 illustrates the EVEREST process, and section **Methods **describes it in detail. This paper describes two runs of the EVEREST process, on Swiss-Prot 40.28 (predating the incorporation of Swiss-Prot into UniProt), and on Swiss-Prot 49.2 (of UniProtKB 7.2).

## Results

**Figure 3:**
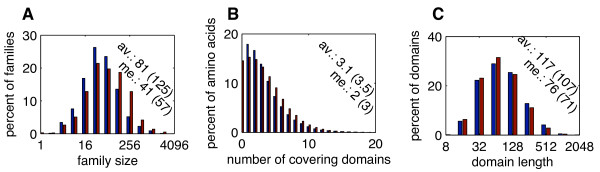
**Statistics of EVEREST domains**. Blue: EVEREST release 1; Red: EVEREST release 2; **av**: average value for release 1 (release 2); **me:**median value for release 1 (release 2). **A: **Distribution of the sizes of domain families (release 1: 1.4 × 10^4 ^families, release 2: 2 × 10^4 ^families, logarithmic bins). **B: **Distribution of the number of domains covering every amino acid. (release 1: 4.2 × 10^7 ^amino acids, release 2: 7.7 × 10^7 ^amino acids, no binning). **C: **Distribution of domain length (release 1: 10^6 ^domains, release 2: 2.5 × 10^6 ^domains, logarithmic bins).

We have first applied EVEREST to Swiss-Prot 40.28, a comprehensive, highly curated, database that includes 114,033 protein sequences. We performed 3 iterations, generating ~100,000 HMMs in the first iteration, ~50,000 HMMs in the second, and ~25,000 HMMs in the third. 13,569 families were defined at the end of the process, to which we refer as EVEREST release 1 families. These families include ~1,000,000 domains, and jointly cover 83% of the amino acids in the Swiss-Prot database. EVEREST release 1 annotates 8816 proteins (8% of the database) that are not annotated by Pfam A. Additionally, in 18,234 proteins (16% of the database), EVEREST annotates a part of the protein that is not annotated by Pfam A. The average (median) size of an EVEREST domain family is 81 (41), the average (median) length of the domains is 117 (76) amino acids. The distributions of family sizes, of domain lengths and of the number of domains covering every amino acid are shown in Figure 3 (blue bars). EVEREST families are frequently variations of known domain families, sometimes adding new domains to the family. Other EVEREST families are new families. Some of these define domains on unannotated parts of proteins. Others identify sub-families, super-families, sub-domains, super-domains or other variations on known domains. We evaluate our results by applying tests to the EVEREST families as described in section **Evaluation of Classification**. A web site providing access to these new domain families is available at [1].

**Figure 4:**
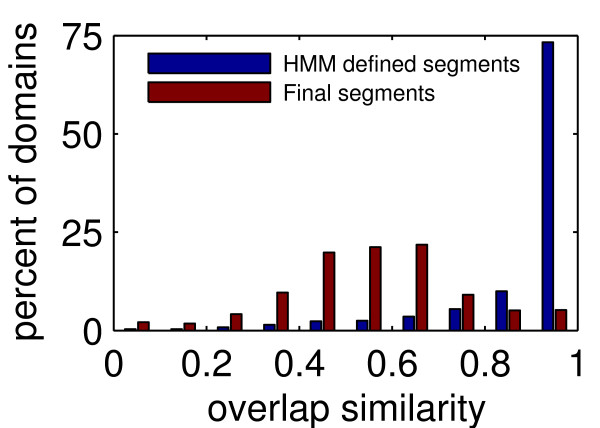
**Redundancy of EVEREST domains**. To evaluate the extent of redundancy of EVEREST domains, we measure, for each domain, its overlap similarity with the most similar other domain on the same protein. We show a histogram over domains of this score. Blue bars – Allowing each HMM of iteration 3 of EVEREST release 1 to define its own domain family (step 7 of the process). Red bars – Final EVEREST release 1 domains, as defined by a vote of sets of HMMs (step 10 of the process).

Since each EVEREST family is defined by its own set of statistical models, different families may intersect each other. To evaluate the extent of this redundancy in the definition of domains, we measure, for each EVEREST domain, its overlap similarity (i.e. the length of the intersection divided by the length of the union) with the most similar other EVEREST domain on the same protein. Allowing each HMM to define its own family results in a significant domain redundancy, as shown by Figure 4 (blue bars). It is not necessarily true that ideal domain families ought to be disjoint, but the extent of the overlap between the families defined by the HMMs appears excessive from a biological perspective. Rather, it appears that for most families, several HMMs have converged each to its own variation of the same domain family. To overcome this problem we identify sets of overlapping HMMs, and replace the families they define with a family defined by a calculated vote. The process is described in detail in steps 9 and 10 of section **The EVEREST Process**. Figure 4 (red bars) shows that nearly all domain redundancy is eliminated by this process.

### Evaluation of classification

We validate our results by comparing the EVEREST families with two reference sets of known families, used as gold standards. The two reference sets we use are Pfam A and SCOP at the level of families (see section **Databases **for details on the databases used).

The definition of an evaluation scheme of a large set of domain families with respect to such gold standards is a complex task, which we have chosen to divide into three tiers. The first two tiers apply to clustering evaluation in general, and not only to evaluation of protein domain classification:

• First, one needs to decide how to compare an evaluated family with a reference family, assuming both group elements of the same universe. We have chosen a standard set similarity measure, namely the ratio between the size of the intersection of the two families and the size of their union.

• Next, one considers comparing an evaluated set of families with a reference set of families, again assuming all families group elements of the same universe. We have chosen a dual view where we check the coverage of the evaluated system by allowing each reference family to select the best fitting evaluated family, and check the accuracy of the evaluated system by allowing each evaluated family to select the best fitting reference family. We describe this in more detail later in this section.

The third tier is specific to the evaluation of systems that identify, and not only classify their elements:

• Recall that EVEREST families classify elements that are distinct from the elements classified by the reference system, i.e. the two sets of families are not defined over the same universe. Rather, each system both defines its own universe of domain instances, and classifies it. We have chosen to project each suggested family to the universe defined by the reference system, thus reducing the problem back to that of evaluation where both systems classify elements of the same universe.

Section **Evaluating a Suggested Domain Family **describes in detail the process of scoring a suggested domain family with respect to a reference family. We refer to this score as *σ *below.

Out of the 13,569 EVEREST release 1 domain families, 12,735 families intersect with Pfam families and 834 do not. 7835 families intersect with SCOP families and 5734 do not. Obviously, EVEREST families that do not intersect with any reference family cannot be evaluated by the reference set. If it turns out that EVEREST families that can be evaluated reconstruct known families well, it will be reasonable to assume that the rest of the EVEREST families are new families of similar quality.

As stated above, we employ two complementary tests:

• Coverage – how many of the reference families are reconstructed well, as described by the histogram over reference families *r *of *σ*(*r*) = max_*e*∈*E*_*σ*(*e, r*), where *E *is the set of EVEREST families.

• Accuracy – how many of the EVEREST families that intersect with the reference families are good reconstructions of any reference family, as described by the histogram over EVEREST families *e *of *σ*(*e*) = max_*r*∈*R*_*σ*(*e, r*), where *R *is the set of reference families.

It is nearly trivial to reconstruct very small families, therefore we only test for coverage of families with at least 5 members (hereafter *non-trivial families*). To test how well EVEREST identifies domain families within a multi-domain context, we also test coverage specifically for families that appear on some protein in a hetero-multi-domain context (hereafter *hetero families*). Reconstructing these families is a much harder task, involving the correct dissection of the protein to its domains. There are 3421 Pfam families of size 5 or more, of which 1764 are hetero families. 383 of the SCOP families have at least 5 members and 166 of those are hetero families.

**Figure 5:**
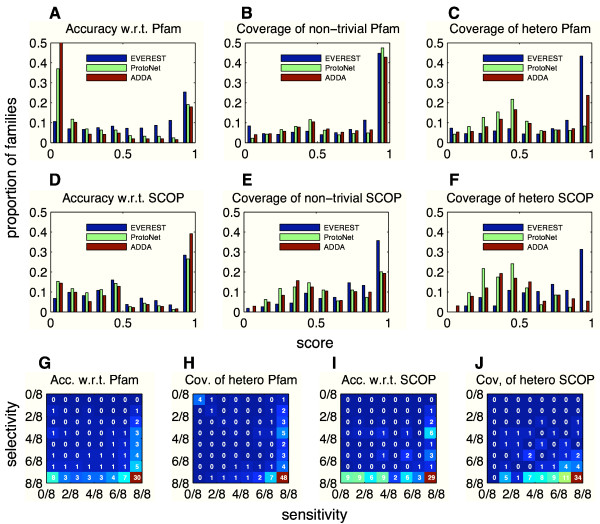
**Performance of EVEREST release 1, ProtoNet and ADDA with respect to Pfam and SCOP. A. Accuracy with respect to Pfam**. Histogram of scores of EVEREST/ProtoNet/ADDA families with respect to best matching Pfam families. **B. Coverage of non-trivial Pfam families: **Histogram over non-trivial Pfam families, of scores of best matching EVEREST/ProtoNet/ADDA families. **C. Coverage of hetero Pfam families: **Histogram over hetero Pfam families, of scores of best matching EVEREST/ProtoNet/ADDA families. **D, E, F: **As A, B, C, with SCOP in place of Pfam. **G. EVEREST Accuracy w.r.t Pfam: **Two-dimensional histogram of sensitivity and selectivity of EVEREST families with respect to best matching Pfam families. **H. EVEREST Coverage of hetero Pfam: **Two-dimensional histogram over hetero Pfam families, of sensitivity and selectivity of best matching EVEREST families. **I, J: **As G, H, with SCOP in place of Pfam. Bins count values above lower threshold, and below or equal to upper threshold (for coverage, Pfam/SCOP families that are not intersected by any EVEREST (ProtoNet/ADDA) family are added to the lowest bin). In **G, H, I **and **J, **numbers are percentages of families in bin. See sections **Evaluation of Classification **and **Evaluating a Suggested Domain Family **for further details.

Figure 5 depicts the accuracy of EVEREST families and coverage of non-trivial reference families and of hetero reference families by EVEREST families (blue bars). Note that EVEREST coverage of the harder case of hetero families is as good as its coverage of all non-trivial families. This is evidence that EVEREST correctly dissects proteins into their domains.

In those instances where EVEREST disagrees with Pfam, it is almost always the case that either EVEREST is highly selective or highly sensitive, as can be seen in panels **G – J **of Figure 5. These are two-dimensional histograms counting the number of families scoring within a given range of selectivity and given range of sensitivity.

It is important to note that the histograms shown underestimate the quality of EVEREST families. Here are two reasons for that:

• **Definition of a domain family is fuzzy. **In some of the cases where EVEREST disagrees with Pfam or SCOP it might be that EVEREST is correct. Section **Examples: Selected EVEREST Families **below lists several such cases.

• **Slightly contaminated new domain families**. We consider those EVEREST families that do not intersect with any Pfam/SCOP family to be putative new domain families. Those families are excluded from the histograms. Consider, however, a novel domain family that is found by EVEREST. If such a family is contaminated by even a single member from a known Pfam/SCOP family, **F**, it would show in the histograms, with a very low score. In this case, our scoring scheme (incorrectly and pessimistically) assumes our new family to be an attempted (poor) approximation of family **F**. Thus are an unknown number of the EVEREST families counted as poor reconstructions of known families, though they are actually good (albeit imperfect) suggestions of honest new families.

We compare our performance to that of two systems with similar goals: the first is ProtoNet -a whole protein sequence hierarchical classification system [20]. ProtoNet was shown to reconstruct protein families to an impressive degree [21]. However, being a whole protein classification it encounters difficulties with multi-domain proteins. The ProtoNet version we have explored clusters the same Swiss-Prot database that we analyze. A recent improvement to ProtoNet has reduced the number of clusters from ~220,000 to 27,823 with nearly no loss in coverage [22]. Of these clusters, 21,829 intersect with Pfam reference families and 6274 intersect with SCOP reference families. The second system is ADDA [12]. This is an algorithm for domain identification and clustering that has significantly improved all previously known methods. ADDA runs over a larger database of ~250,000 sequences, and yields 202,427 families of which 15833 intersect with Pfam reference families and 2427 intersect with SCOP families.

Figure 5 depicts the performance of ProtoNet (green bars), and ADDA (red bars). We have also evaluated a sub-collection of ADDA families, namely those families of size at least 5. While this greatly increases ADDA accuracy, it also further reduces its coverage (not shown).

EVEREST always achieves better coverage, for both Pfam and SCOP, than the other systems. ADDA outperforms EVEREST in terms of accuracy with respect to SCOP. Note also, that unlike EVEREST, the other systems suffer a large reduction of coverage on hetero Pfam/SCOP families.

Table 1 summarizes the data of the analysis of EVEREST release 1, ProtoNet and ADDA with respect to Pfam. For various combinations of sensitivity and selectivity thresholds, the table lists, under *Accuracy*, the percentage of EVEREST, ProtoNet and ADDA families that pass the thresholds with respect to some Pfam family, and under *Hetero Family Coverage *the percentage of hetero Pfam families with respect to which some EVEREST, ProtoNet or ADDA family passes the thresholds. It can be clearly seen that EVEREST outperforms both ProtoNet and ADDA in terms of both accuracy and coverage, for all threshold combinations checked.

To verify that we have not gained knowledge on specific families from our training set, we look at the distribution of scores for the training families and for the non-training families separately. These results are also shown in Table 1, and exhibit very little difference between the two distributions.

### Examples: selected EVEREST families

As shown in Figure 5, many of the EVEREST families are near-perfect reconstructions of known Pfam and SCOP families, scoring high in our tests. The examples in this section are not of those high scoring families, rather, we consider several families that do not score high with respect to Pfam. As noted above, some of these families provide a different, valid, interpretation of the sequence data. Others might be lower quality versions of the Pfam families, which, nevertheless, provide clues through which, either by manual inspection or by further development of the algorithm, one can identify the biologically genuine domain family.

Because they do not agree with Pfam, these families achieve low scores in the tests reported, providing evidence that the tests should be considered lower bounds on the quality of EVEREST.

#### Functional annotation for a family with unknown function

**Figure 6:**
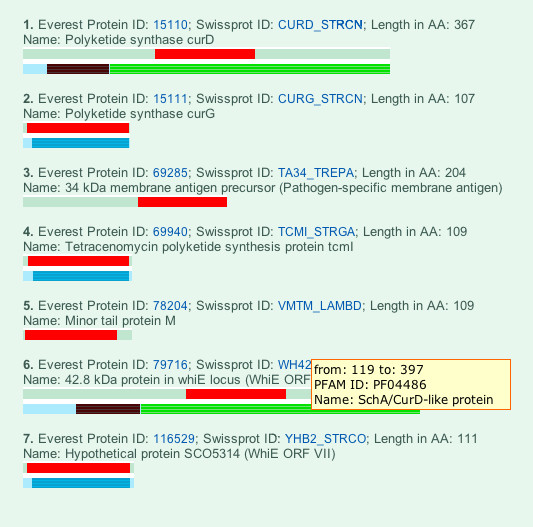
**EVEREST family EV01.01017**. Excerpt from the EVEREST web page for [EVEREST:EV01.01017]. Seven representative proteins (out of eight) containing domains of [EVERERST:EV01.01017] are illustrated. Red segments illustrate [EVEREST:EV01.01017] domains, green segments illustrate [Pfam:PF04486] domains, blue segments illustrate [Pfam:PF04673] domains and brown segments illustrate [Pfam:PF03992] domains. For details of this example see **Functional annotation for a family with unknown function **in section **Examples: Selected EVEREST Families**.

[EVEREST:EV01.01017] is composed of all the domains of [Pfam:PF04673] (Polyketide synthesis cyclase), the middle part of all the domains of [Pfam:PF04486] (SchA/CurD like protein) and two more unannotated domains. According to Pfam, SchA/CurD like family has no known functional role, but two of its members are known to be part of gene clusters involved in the synthesis of polyketide-based spore pigments. We therefore find it possible that those two families should actually be considered one, as suggested by our system. Figure 6 illustrates the [EVEREST:EV01.01017] page from the EVEREST web server.

#### Putative new domain family

**Figure 7:**
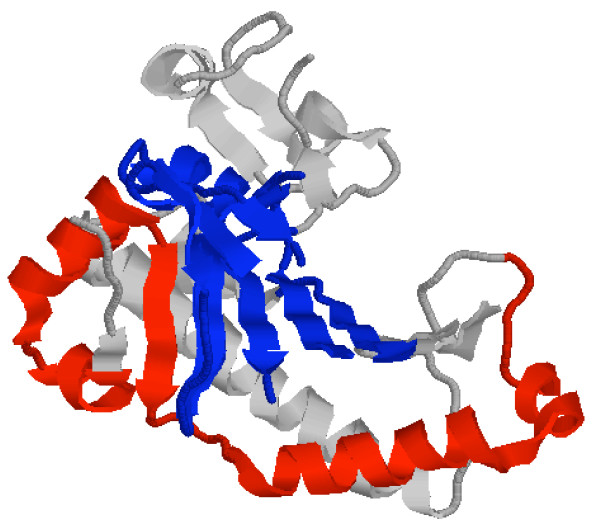
**EVEREST family EV01.02755**. [PDB:1UOG], a representative protein structure containing an [EVEREST:EV01.02755] domain (in red) and a [Pfam:PF03171] domain (in blue). Notice that [EVEREST:EV01.02755] defines a domain distinct from [Pfam:PF03171]. For details of this example see **Putative new domain family **in section **Examples: Selected EVEREST Families**.

[EVEREST:EV01.02755] is unknown to Pfam – none of its domains intersect with any Pfam domain. We hypothesize that it is a new domain family. Out of the 55 domains in [EVEREST:EV01.02755], 54 appear N-terminal to domains of [Pfam:PF03171] (2OG-Fe(II) oxygenase superfamily), with a gap of about 90 amino acids between them. We consider the consistent appearance of [Pfam:PF03171] C-terminal to [EVEREST:EV01.02755] domains as supporting evidence to the hypothesis that [EVEREST:EV01.02755] is a new domain family. Figure 7 illustrates a representative protein structure containing [EVEREST:EV01.02755] and [Pfam:PF03171].

#### Sub-families

**Figure 8:**
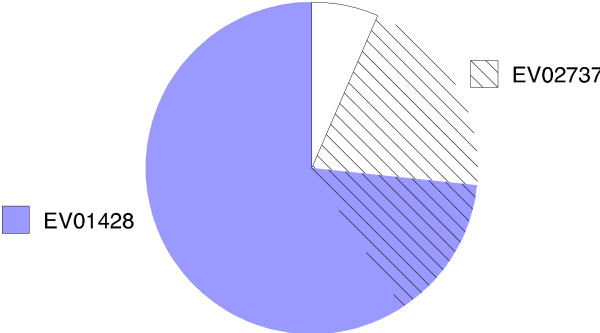
**Coverage of [Pfam:PF00047] by [EVEREST:EV01.01428] and [EVEREST:EV01.02737]**. Blue area – proportion of [Pfam:PF00047] domains covered by [EVEREST:EV01.01428]. Striped area -proportion of [Pfam:PF00047] domains covered by [EVEREST:EV01.02737]. Together, both EVEREST families cover 94% of [Pfam:PF00047]. For details of this example see **Sub-families **in section **Examples: Selected EVEREST Families**.

[Pfam:PF00047] (immunoglobulin-like), contains 1976 domains in our database. It is found in hundreds of different domain contexts. Of its 1976 domains, 1451 are found by [EVEREST:EV01.01428], which also introduces 7 false positives and 133 unannotated domains (yielding a score of 0.73). No other EVEREST family achieves higher coverage of [Pfam:PF00047]. Identifying two EVEREST domains if their intersection is at least 80% of their union, we have 26 other EVEREST families that intersect with [EVEREST:EV01.01428]. Of those, the intersection of [EVEREST:EV01.02737] with [EVEREST:EV01.01428] covers 20% of [EVEREST:EV01.01428] and 44% of [EVEREST:EV01.02737]. [EVEREST:EV01.02737] is the only family that is a good candidate for complementing [EVEREST:EV01.01428] in the coverage of [Pfam:PF00047], since on the one hand, their intersection is not trivial, and on the other hand, neither one covers the other. [EVEREST:EV01.02737] finds 683 of the 1976 domains of [Pfam:PF00047], in addition to 17 false positives and 52 unannotated domains (yielding a score of 0.34). When taken together [EVEREST:EV01.01428] and [EVEREST:EV01.02737] find 1850 of [Pfam:PF00047] domains, and introduce 22 false positives (which would have resulted in a score of 0.93). Figure 8 represents the coverage of [Pfam:PF00047] by [EVEREST:EV01.01428] and [EVEREST:EV01.02737].

#### Super-family

**Figure 9:**
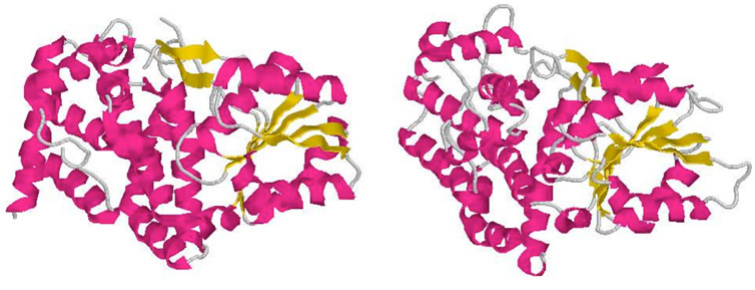
**EVEREST family EV01.04463**. Left: [PDB:1JQA], a representative protein structure of SCOP family "Iron-containing alcohol dehydrogenase". Right: [PDB:1DQS], a representative protein structure of SCOP family "Dehydroquinate synthase, DHQS". Both families belong to SCOP superfamily "Dehydroquinate synthase-like", which is fully reconstructed by [EVEREST:EV01.04463]. Notice how the two structures are distinct, yet similar. For details of this example see **Super-family **in section **Examples: Selected EVEREST Families**.

[EVEREST:EV01.04463] fully covers both [Pfam:PF00465] (Iron-containing alcohol dehydrogenase, 54 domains) and [Pfam:PF01761] (3-dehydroquinate synthase, 36 domains), and contains no other Pfam domains, therefore its score is 0.6 with respect to [Pfam:PF00465] and 0.4 with respect to [Pfam:PF01761]. Taking a closer look into those two families, one observes that ENZYME [23] classifies [Pfam:PF00465] to EC 1.1, while [Pfam:PF01761] is sometimes classified to EC 4.6 and other times to EC 1.1. The SCOP family corresponding to [Pfam:PF00465] is "Iron-containing alcohol dehydrogenase" ([SCOP:69892]) while [Pfam:PF01761] corresponds to SCOP family "Dehydroquinate synthase, DHQS" ([SCOP:56797]). Together, these two families form SCOP superfamily "Dehydroquinate synthase-like" ([SCOP:56796]), thus [EVEREST:EV01.04463] reconstructs a known super-family. Figure 9 shows representative protein structures from each one of the two SCOP families.

#### Domain cis-combinations

[Pfam:PF00595] (PDZ domain) is a relatively common domain family, appearing 229 times in Swiss-Prot 40.28, in several different domain contexts. [EVEREST:EV01.12145] finds 213 out of these 229 occurrences, adding 30 false positives (score 0.82). [Pfam:PF00640] (Phosphotyrosine interaction domain) appears 44 times in Swiss-Prot 40.28, and is fully reconstructed, with no false positives by [EVEREST:EV01.01420] (score 1). The combination of a [Pfam:PF00640] domain followed by two [Pfam:PF00595] domains appears 9 times in Swiss-Prot 40.28. [EVEREST:EV01.09528] finds all of these 9 times with no false positives.

### EVEREST release 2

We have run EVEREST on an up-to-date version of Swiss-Prot 49.2 (UniProtKB 7.2). Again we have performed 3 iterations, generating ~150,000 HMMs in the first iteration, ~75,000 HMMs in the second and ~37,500 HMMs in the third. 20,230 families defined at the end of the process form EVEREST release 2. The red bars in Figure 3 show statistics of EVEREST release 2 families. Note that these have not changed much from the statistics of release 1.

The training data used for this run was a taken from a up-to-date version of Pfam (release 19.0). We have analyzed the performance of EVEREST release 2 with respect to both this Pfam release and the Pfam release that was used in EVEREST release 1 (Pfam release 9). Figure 15 compares the performance of EVEREST release 2 with that of release 1. Notice that the accuracy of both EVEREST releases with respect to both Pfam releases does not change (panel **A**). Note also that the EVEREST release 2 has better coverage of Pfam release 9, but that Pfam release 19 is a harder reference set to cover (panels **B **and **C**). Notice also, that as in release 1, the coverage of Pfam by EVEREST does not drop when focusing on hetero families.

Figure 15 also depicts performance with respect to SCOP. The comparison between the performance of the two EVEREST releases is only qualitative for the two following reasons: The first is that each EVEREST release is measured against a different SCOP release. The second reason is a change in the methodology of comparison. Whereas for release 1, the EVEREST domains were mapped from the Swiss-Prot sequences to the PDB sequences (see **Databases), **we have used a different technique with release 2. The HMMs and HMM sets definitions of EVEREST release 2 families were used to scan all PDB sequences, defining EVEREST domains directly on PDB sequences, thus avoiding the need to map EVEREST domains from Swiss-Prot to PDB sequences.

Accepting 75% selectivity combined with 75% sensitivity as a good reconstruction of a family, we are able to reconstruct 56% of the hetero Pfam families and 63% of the hetero SCOP families. Further more, since 51% of our domain families that intersect with Pfam of SCOP are good reconstructions of either a Pfam family or a SCOP family, we can assume that 51% of our 1000 domain families that do not intersect with Pfam or SCOP are good suggestions of new families.

## Discussion

33% of the Swiss-Prot proteins (release 40.28 of UniProtKB 7.2) that are annotated by Pfam (release 19.0) contain more than one Pfam domain. This number is clearly an underestimate of the proportion of multi-domain proteins since for many proteins Pfam identifies but one of several domains. The abundance of multi-domain proteins is one of several indications that it is important to develop tools to investigate proteins at the level of their domains.

One finds in the literature a number of definitions for the concept of a protein domain. The approach we have taken is evolutionary. We define a domain as a continuous sequence of amino acids that recurs (non trivially) in the protein space. It should be noted that the most widely accepted definition of domain is based on a structural perspective. Recall that EVEREST uses no structural data whatsoever. Thus, it is rather surprising that EVEREST reconstructs 63% of SCOP domain families to a high degree. However, EVEREST does well not only according to the structural definition. Some of our families correspond to sub-domains, at least 400 of the EVEREST families correspond to cis-combinations (super-domains) of Pfam and SCOP families (such as the one reported under **Examples: Selected EVEREST Families**),and others describe other situations. We believe that interesting phenomena appear in a range of segment lengths, and that the relationships between recurring patterns of different lengths can teach us about the biology behind the sequence.

We have compared our results to those of ADDA. A problematic aspect of this comparison is that ADDA was run on a larger database than the one we used. It is possible that our evaluation of ADDA's performance vs. Pfam is hampered by the mapping down to the Swiss-Prot database. This issue does not arise with our analysis of ADDA's performance vs. SCOP, since there both systems were mapped to the PDB sequence database.

We use a combination of methods from different disciplines to iteratively define domain families and select the best among them. This allows us to achieve high coverage and accuracy in the families we define. We believe the methodologies we employ here may be of independent interest. Whenever one studies similarity relations, arises the challenge of deriving the *correct *transitive closure. Some of the ideas developed here seem applicable in this much wider context. We had to deal with another commonly occurring notoriously difficult problem, namely, when to stop an ongoing clustering process. Our algorithm constructs a comprehensive hierarchy of clusters and proceeds to weed it using machine learning methods. Again this approach may help solve this problem in other situations.

A challenging problem which we did not thoroughly study here is that of determining the exact location of our domains' boundaries. We intend to tackle this problem with a procedure for constructing HMMs which can extend or reduce the HMM according to information-theoretic criteria.

Interesting phenomena show up upon investigating the relationships between EVEREST families (as well as between EVEREST, Pfam and SCOP families). Some pairs of families exhibit a sub-domain – super-domain relationship, other are sub-family – super-family pairs, yet others appear side-by-side, etc. We plan to rigorously define and investigate such relationships, providing an additional layer to EVEREST, on top of the definition of families.

Finally, we have created a web site for EVEREST. It allows browsing through EVEREST domain families, providing views for the domains found on a requested protein, and for the domains of a requested family (within their protein contexts). In the future, this site will also offer tools to explore relationships between different families, etc. We hope the web site will be useful for various types of research in biology.

## Conclusion

The main achievement that we report here is the development of an automatic method to identify and classify protein domains based on sequence data. The whole process, called EVEREST, was applied to the Swiss-Prot database. EVEREST domains cover 85% of the amino acids in the database. EVEREST finds domains in 11,852 proteins (6% of the database) that are not annotated by Pfam A. In additional 43,086 proteins (20% of the database), EVEREST finds domains in regions that are not annotated by Pfam A.

We evaluate EVEREST by testing how well it reconstructs "gold standard" domain families taken from Pfam A and SCOP. The results show that EVEREST reconstructs 56% of the Pfam A families and 63% of the SCOP families, and that 51% of the EVEREST families are good reconstructions of either Pfam A families or SCOP families. Manual review of EVEREST families that do not score well with respect to any known family, suggests that many of them do determine valid domain families that are either unknown to Pfam and SCOP, or are valid alternatives to their definitions.

We believe EVEREST provides three contributions to our understanding of the protein world. The first is its annotation of previously un-annotated proteins or regions of proteins. The second is its novel unbiased view of domain families: as shown in section **Examples: Selected EVEREST Families, **many of the EVEREST families that do not technically agree with Pfam or SCOP families provide valid, complementary interpretations of the biological data. The third contribution is a promise – Being an automatic process, EVEREST is scalable. While not a trivial task, it is clearly possible to run EVEREST on larger databases, and we will do that in the near future. After having proven the ability of the process to define high quality protein domain families on the Swiss-Prot section of the UniProt Knowledgebase, we expect to provide such results for the whole of UniProt, greatly increasing the proportion of annotated protein regions.

## Methods

### Databases

#### Inputs to the EVEREST process

Swiss-Prot release 40.28 was the source of the protein sequences for our EVEREST release 1, where as Swiss-Prot release 49.2 (of UniProtKB 7.2) was the source for EVEREST release 2. Pfam A release 9.0 domains were taken from InterPro release 6.2 (for EVEREST release 1) [24] (as defined in the file protein2interpro.dat downloaded from ). We used all and only those Pfam domains that are defined on Swiss-Prot release 40.28 proteins. EVEREST release 2 was built and analyzed using Pfam A release 19.0 from InterPro release 12.1.

We use only Pfam A families since those are high quality, manually curated families, while Pfam B provides a set of automatically defined families of unknown quality. A random half of Pfam A families was used as training set for the EVEREST process.

#### Competitor systems

The ProtoNet version we have analyzed was run on the same Swiss-Prot database, release 40.28. The ADDA sequence database and the ADDA domain classification were downloaded from the ADDA server on November 2003.

#### Evaluation reference systems

Pfam domains were downloaded as described above. For the analysis of EVEREST release 1, SCOP domains were taken from ASTRAL release 1.61 [25] (with a similarity cutoff of 95%). We employed the following procedure in order to map the SCOP domains to Swiss-Prot proteins:

• All SCOP domains that are not continuous in sequence were removed.

• The start and end positions of each SCOP domain within the relevant PDB sequence were identified.

• Each PDB sequence was aligned with the best matching Swiss-Prot release 40.28 sequence (for testing EVEREST and ProtoNet), and with the best matching ADDA database sequence (for testing ADDA).

• When assessing the quality of a suggested domain family with respect to SCOP, the domains of the family were first projected, using the above alignments, from their definition on Swiss-Prot (for EVEREST and ProtoNet families) or the ADDA database (for ADDA families), to the matching PDB sequence(s). See section **Evaluating a Suggested Domain Family **for further details.

For the analysis of EVEREST release 2, SCOP domains were taken from ASTRAL release 1.69, again with a similarity cutoff of 95%, and discarding all domains that are not continuous in sequence. Since EVEREST release 2 family definitions were used to directly scan the PDB sequences, there was no need to map PDB sequences to Swiss-Prot sequences here.

### The EVEREST process

Following is a description of the EVEREST process. Each step is illustrated by an arrow in Figure 2.

**Figure 10:**
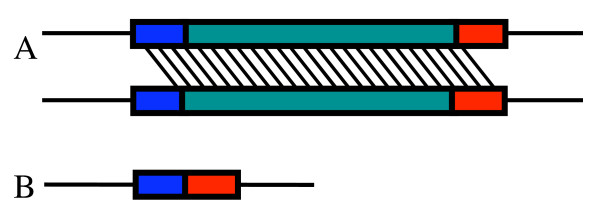
**A schematic representation of the repeat removal process**. **A**. Two copies of the protein are shown. Colored area indicates a repeat region. Blue marks the first repeat unit, orange marks the last and green the rest. The diagonal lines mark the alignment that matches the repeat region to itself. The first repeat unit starts with the first aligned position and ends just before the position it aligns to. The last repeat unit ends with the last position aligned and begins right after the position it aligns to. **B**. The representation of the new sequence, after all but the first and last repeat units are "spliced out". For an extreme example, protein [Swiss-Prot:P08519] that contains 38 units of the Kringle domain, is reduced from 4548 amino acids to 467 amino acids following this step.

0. The input to the algorithm is a full database of protein sequences (represented by panel **A**). (Swiss-Prot 40.28 containing ~114,000 sequences for EVEREST release 1, Swiss-Prot 49.2 containing ~211,000 sequence for EVEREST release 2). [26].

1. A non-redundant database (panel **B**) is created from the input database. We run BLAST [3] and compare every protein in the database with every other protein. We allow a protein to represent another provided that their BLAST similarity score is very significant (E- score < 1*e *- 90) and their BLAST alignment covers at least 95% of each one of them. We create a non-redundant database by applying a greedy algorithm to find an inclusion-minimal dominating set in the appropriate graph. Every protein in **A **has at least one representative protein in **B**, and no two proteins in **B **represent each other. The resulting non-redundant database contains ~72,000 sequences for EVEREST release 1 and ~125,000 sequences for EVEREST release 2.

2. It quickly turns out that proteins containing regions of three or more consecutive repeated segments can lead to numerous false conclusions. To discover such repeating regions, we compare every protein from **B **to itself. This is carried out using an iterative variation on the Smith-Waterman sequence comparison algorithm [27]. At each iteration the protein at hand is matched against itself subject to the condition that positions that were matched in previous iterations cannot be matched. An alignment between two overlapping (non-identical) segments is interpreted as indicating a repeated region in the sequence. We remove all but the first and the last repeating units of the repeated region, and reiterate the program to seek other repeated regions in the protein at hand. See Figure 10 for a schematic description of the process. Panel **C **represents the database of representative sequences following the removal of internal repeats. For EVEREST release 1, out of the ~72,000 sequences, ~9,000 were found to contain repeated regions (~15,000 out of ~125,000 for release 2).

3. Using the same BLAST run from step 1, we compose a list of possibly-similar pairs of proteins by setting a very relaxed threshold on the BLAST score (E-score < 100). We then apply the above variant of the Smith-Waterman algorithm to each pair in the list. Segments which are found to be significantly similar to other segments are collected into a segment database (panel **D**). Each segment in the database has another segment paired with it – the one it was found to be similar to (dashed blue lines in panel **D **represent such pairings).

We now have a database of segments (putative domains), with two similarity measures defined upon them:

• The sequence similarity between a segment and its mate.

• An overlap similarity between every two segments on the same protein. Namely, the length of their intersection divided by the length of their union.

For EVEREST release 1, ~23,000,000 segments were created (~51,000,000 for release 2).

4. The segments on each protein are clustered into groups according to their overlap similarity. Panel **E **represents the database of groups created in this stage. The sequence similarity of the segments is inherited by their groups, thus if group *α *contains a segment that is similar to a segment in group *β*, then there is a sequence similarity edge between *α *and *β *(represented by a dashed blue line).

We use a very conservative clustering algorithm at this stage, and require every two segments that are in the same group to have overlap similarity of at least 0.5. This is a powerful filter against false transitivity induced by sequence similarity edges.

**Figure 11:**
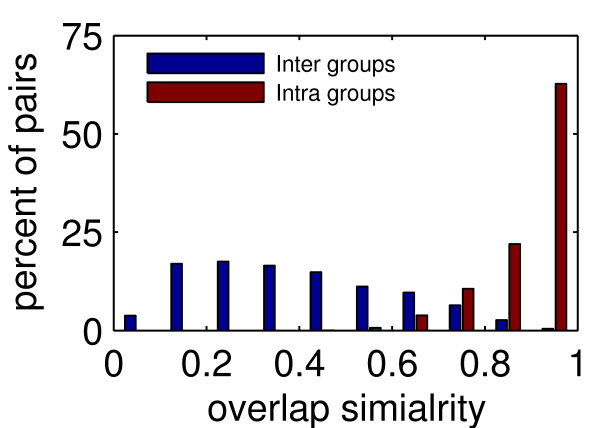
**Distribution of overlap similarities**. Overlap similarities between pairs of segments generated in step 3 (first iteration) of EVEREST release 1. Two sets of pairs of segments are observed. The first (blue) contains all pairs of segments where the two segments of the pair are on the same protein, but clustered into different groups in step 4. The second (red) contains all pairs of segments where both segments of the pair are clustered into the same group. Histograms of overlap similarities of the segment pairs of the two sets are shown.

Figure 11 depicts the distribution of segment overlap similarities intra-groups and inter-groups for the first iteration of EVEREST release 1. The strict algorithm we use in this step can assign two segments with high overlap similarity to two different groups. As shown by the figure, this rarely occurs, a good indication for the validity of our clustering procedure.

For EVEREST release 1, ~2,000,000, ~1,200,000 and ~1,000,000 groups were created in the first, second and third iteration respectively (~3,300,0000, ~2,700,000 and ~2,500,00 for release 2).

5. The groups from **E **are clustered according to their sequence similarity, using an average linkage algorithm: Let *σ*(*α*, *β*) denote the sequence similarity between group *α *and group *β *(assigning a default value to every pair of groups that share no similar segments). Then the similarity between two clusters of groups *C *and *D *is defined as *σ*(*C, D*) = 1|C||D|
 MathType@MTEF@5@5@+=feaafiart1ev1aaatCvAUfKttLearuWrP9MDH5MBPbIqV92AaeXatLxBI9gBaebbnrfifHhDYfgasaacH8akY=wiFfYdH8Gipec8Eeeu0xXdbba9frFj0=OqFfea0dXdd9vqai=hGuQ8kuc9pgc9s8qqaq=dirpe0xb9q8qiLsFr0=vr0=vr0dc8meaabaqaciaacaGaaeqabaqabeGadaaakeaadaWcaaqaaiabigdaXaqaamaaemaabaGaem4qameacaGLhWUaayjcSdWaaqWaaeaacqWGebaraiaawEa7caGLiWoaaaaaaa@3610@ ∑_*α*∈*C*,*β*∈*D*_*σ*(*α*, *β*). We start with a singleton cluster for each group, and iteratively merge the two most similar clusters until we are left with one cluster. We keep a record of every cluster we create during this process. Panel **F **represents the hierarchy of clusters created. The leaves of the tree correspond to the groups from **E**.

Consider all the segments on a certain protein that belong to a specific cluster. It would seem reasonable to allow these segments collectively to define a domain. This, however, is clearly incorrect for homo-multi-domain proteins where it is necessary to initially discern the (multiple) occurrences of the same pattern. We do that by identifying connected components in the graph of overlap similarities defined over the segments of the protein in the cluster. Each such connected component defines a domain in the family. The boundaries of the domain are defined by taking the 40th percentile from outside of the boundaries of the segments in the connected component.

Since the hierarchy of clusters is a binary tree, the number of clusters created (including singletons) is twice the number of groups in **E**.

The blue bars in panels **A **and **B **of Figure 14 show the performance of the candidate domain families created in this stage in iteration 1 of EVEREST release 1.

6. Each cluster in **F **is a candidate family. Most of these are inappropriate and should be discarded. This is carried out using machine learning techniques to sift through these families:

We use a randomly chosen set of half of the Pfam families as a training set. For each cluster, we calculate its score according to the training set, as described in section **Evaluating a Suggested Domain Family**. We also calculate a set of intrinsic features, independent of any Pfam knowledge (similarity of the two clusters that merged into it, cluster size, similarity within cluster, variance of length of the domains in cluster, etc.).

We use a boosting algorithm [17] to learn a regression function from the intrinsic features to the score of a cluster. The scores assigned by the regression function are used as a guideline for selecting a set of representatives of the "good" clusters in the hierarchy. Two observations direct our choice: First, the hierarchy contains many "bad" clusters, that we wish to eliminate. Second, due to the gradual nature of the clustering algorithm, the hierarchy it creates essentially contains many near duplicates – Clusters that are very similar to each other. Often, cluster *C *that is created by merging *A *and *B *is similar to *A *(or *B*). If *C *is "good", then *A *is also likely to be good. However, both of them would be good with respect to the same domain family, and selecting both of them would be redundant. Therefore, we should not select two clusters where one is an ancestor of the other unless they significantly differ in size. We use a greedy algorithm to pick the clusters, taking each time the highest scoring cluster that is allowed. The circles in panel **G **represent the clusters that are chosen in this procedure.

For EVEREST release 1, we pick 100,000, 50,000 and 25,000 clusters in the first, second and third iteration respectively (150,000, 75,000 and 37,500 for release 2).

**Figure 12:**
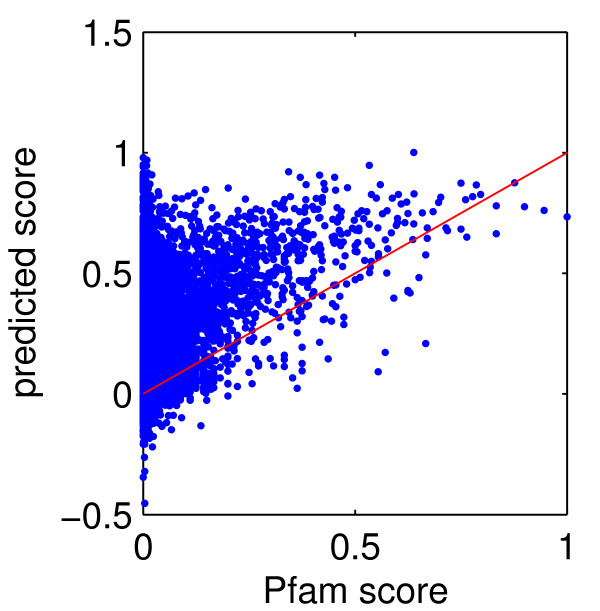
**Performance of the regression function in iteration 1**. Data points correspond to a random set of ~13,000 of the clusters created in step 5 of iteration 1 of EVEREST release 1, and intersecting with Pfam. **x-axis: **score of the cluster according to Pfam. **y-axis: **score of cluster according to the regression function learned in step 6 of iteration 1. See section **Evaluating a Suggested Domain Family **for the definition of a domain family score.

Figure 12 compares the scores assigned by the regression function of iteration 1 to a random set of families with their actual scores. Note a distinct tail of families with very low Pfam scores, but high scores by the regression function. These might be good families that are missing from the current Pfam.

The green bars in panels **A **and **B **of Figure 14 show performance of the domain families selected in this stage in iteration 1 of the run generating EVEREST release 1. One can see that the accuracy of the selected families is much better than the accuracy of the general population of clusters from step 5, while the reduction in coverage is small.

7. We construct an HMM for each cluster chosen in **G**, using Clustal-W (ver. 1.8) [28] and HMMER (ver. 2.3.2) [18]. Because Clustal-W cannot align large numbers of sequences, at most 100 (randomly selected) sequences from each cluster are input to Clustal-W. An HMM is constructed for this reduced alignment (hmmbuild program), and used to align all the sequences in the cluster (hmmalign program). Then the HMM is recalculated using the new alignment (hmmbuild program), and calibrated (hmmcalibrate program). hmmbuild, hmmalign and hmmcalibrate are programs within the HMMER package. Default parameters are used for all programs.

For a small fraction of the clusters, Clustal-W crashes, no HMM is created, and the family is discarded.

8. We recreate the segments database of **D **by scanning the original database **A **using each of the HMMs from **H**. We use a threshold of E-score < 1 to define the segments. To reduce the running time of scanning an order of 10^5 ^sequences with and order of 10^5 ^HMMs, we have developed an acceleration scheme for HMMER [29].

To complete the definition of the segment database, we need to define two similarity measures between segments, as in step 3.

• The sequence similarity between every two segments created by the same HMM is defined to be the sum of the E-scores of their creation.

• As before, we define the overlap similarity of two segments on the same protein as the length of their intersection divided by the length of their union.

With the newly recreated segment database in hand, we can reiterate steps 4–8.

The segments found by each HMM are a suggested domain family. The red bars in panels **A **and **B **of Figure 14 show the performance of those domain families in this stage in iteration 1 of the run generating EVEREST release 1. The increase in the quality of the families due to the introduction of HMMs, both in terms of accuracy and in terms of coverage, is evident. The blue and green bars in panels **C **and **D **show the performance of the families defined by the HMMs in iterations 2 and 3 of the run generating EVEREST release 1.

9. As seen in Figure 4 (blue bars), most of the segments, are found by more than one HMM. This leads to a significant redundancy in the domains defined. To overcome this redundancy, we identify overlapping families and merge them, as described below.

First we define and calculate the overlap between two HMM's from iteration 3: the process is continued through step 4 once more (except the overlap similarity threshold is set at 0.7 instead of 0.5). Then we associate each HMM with the set of groups from step 4 that contain segments defined by the HMM. The overlap between two HMMs is defined as the intersection of their associated sets.

**Figure 13:**
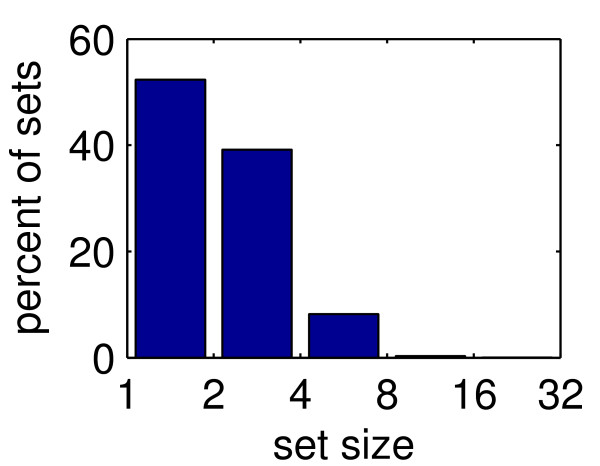
**Sizes of HMM sets**. Distribution of number of HMMs per HMM set (for EVEREST release 1). See step 9 of section **The EVEREST Process **for details.

We now find sets of HMMs where each member of the set overlaps by at least an 18
 MathType@MTEF@5@5@+=feaafiart1ev1aaatCvAUfKttLearuWrP9MDH5MBPbIqV92AaeXatLxBI9gBaebbnrfifHhDYfgasaacH8akY=wiFfYdH8Gipec8Eeeu0xXdbba9frFj0=OqFfea0dXdd9vqai=hGuQ8kuc9pgc9s8qqaq=dirpe0xb9q8qiLsFr0=vr0=vr0dc8meaabaqaciaacaGaaeqabaqabeGadaaakeaadaWcaaqaaiabigdaXaqaaiabiIda4aaaaaa@2EAA@th of its size with every other member. The sizes of the sets are shown in Figure 13. To test that the sets of HMMs are homogeneous, i.e. within each set all HMMs describe the same family, we looked at the Pfam families associated with the HMMs of each set. We labeled each HMM scoring at least 0.5 with the Pfam family it best matches. For EVEREST release 1, out of the 3830 sets where more than one HMM was labeled, in 3615 (94%) sets all HMMs were labeled by the same Pfam family.

10. We wish to define a domain family per HMM set found in step 9. To that end, we convert our HMMs from the default global-local mode, where the alignment is global in the HMM and local in the searched sequence, to global-global mode, where the alignment is required to account for all the sequence. We then score each segment found by each HMM in the set, using the global-global version of all HMMs in the set, and take the average E-score. Segments for which this average score is at most 1 are included in the family. As in step 5, we might now have several segments that are variations of the same domain within the family. We employ the same process used there for merging these segments and defining domain boundaries.

**Figure 14:**
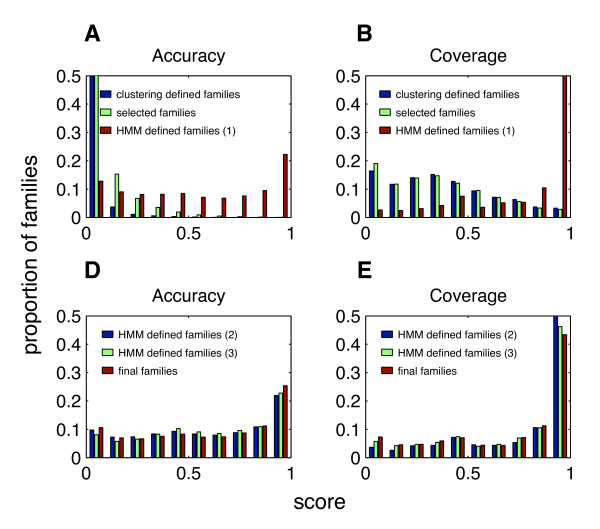
**Performance along the EVEREST process generating release 1 with respect to Pfam**. **A **and **B**: Blue bars – domain families defined in the clustering process of step 5 in the first iteration; green bars – selected domain families chosen in step 6 in the first iteration; red bars – domain families defined by the HMMs of step 7 in the first iteration. **C **and **D**: Blue bars – domain families defined by the HMMs of step 7 in the second iteration; green bars – domain families defined by the HMMs of step 7 in the third iteration; red bars – domain families defined by voting of sets of the HMMs of step 7 in the first iteration. Coverage shown is of hetero Pfam families. See sections **Evaluation of Classification **and **Evaluating a Suggested Domain Family **for further details.

**Figure 15:**
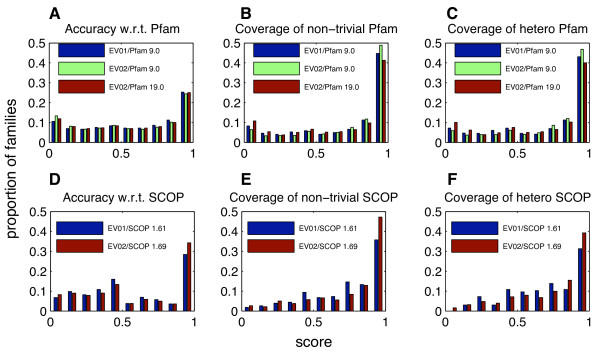
**Performance of EVEREST release 1 and EVEREST release 2 with respect to Pfam and SCOP**. **EV01**: EVEREST release 1; **EV02: **EVEREST release 2. **A. Accuracy with respect to Pfam: **Histogram of scores of EVEREST families with respect to best matching Pfam families. **B. Coverage of non-trivial Pfam families: **Histogram over non-trivial Pfam families, of scores of best matching EVEREST families. **C. Coverage of hetero Pfam families: **Histogram over hetero Pfam families, of scores of best matching EVEREST families. **D, E, F: **As A, B, C, with SCOP in place of Pfam. See sections **Evaluation of Classification, EVEREST Release 2 **and **Evaluating a Suggested Domain Family **for further details.

The domain families defined in this stage are the final output of our process. Their quality is discussed in section **Results**. For convenience of comparison, their performance is also depicted by the red bars of panels **C **and **D **of Figure 14.

### Evaluating a suggested domain family

Following is a procedure for evaluating a cluster. It is used whenever a suggested domain family is evaluated against a reference set of domain families.

The procedure uses a set of known domain families as reference. In stage 6 the reference set is the training set of Pfam families defined there. In section **Results**, all Pfam families and all SCOP families are independently used as reference sets.

We define ∏(*s*), the *reference projection *of a suggested family *s *as the set of reference domains that significantly intersect with the domains of the suggested family. A reference domain and a cluster domain are said to be significantly intersecting if their intersection is at least 80% of the shorter of the two. Suggested families whose reference projection is empty cannot be evaluated by the reference set, and are ignored.

For some tests the above definition implies that we first map the domains of the suggested family to the sequences upon which the reference set is defined using pre-calculated pairwise alignments between each target sequence and the most similar sequence in the source sequence database. This mapping was applied for the analysis of EVEREST release 1, ADDA and ProtoNet with respect to SCOP, and for the analysis of ADDA with respect to Pfam.

We are now able to define parameters for comparison between a suggested family *s *and a reference family *r*.

selectivity:φ(s,r)=|Π(s)∩r||Π(s)|     (1)
 MathType@MTEF@5@5@+=feaafiart1ev1aaatCvAUfKttLearuWrP9MDH5MBPbIqV92AaeXatLxBI9gBaebbnrfifHhDYfgasaacH8akY=wiFfYdH8Gipec8Eeeu0xXdbba9frFj0=OqFfea0dXdd9vqai=hGuQ8kuc9pgc9s8qqaq=dirpe0xb9q8qiLsFr0=vr0=vr0dc8meaabaqaciaacaGaaeqabaqabeGadaaakeaafaqabeqacaaabaacbaGae83CamNae8xzauMae8hBaWMae8xzauMae83yamMae8hDaqNae8xAaKMae8NDayNae8xAaKMae8hDaqNae8xEaKNae8NoaOdabaacciGae4NXdyMaeiikaGIaem4CamNaeiilaWIaemOCaiNaeiykaKIaeyypa0ZaaSaaaeaadaabdaqaaiabfc6aqjabcIcaOiabdohaZjabcMcaPiabgMIihlabdkhaYbGaay5bSlaawIa7aaqaamaaemaabaGaeuiOdaLaeiikaGIaem4CamNaeiykaKcacaGLhWUaayjcSdaaaaaacaWLjaGaaCzcamaabmaabaGaeGymaedacaGLOaGaayzkaaaaaa@5B69@

sensitivity:ψ(s,r)=|Π(s)∩r||r|     (2)
 MathType@MTEF@5@5@+=feaafiart1ev1aaatCvAUfKttLearuWrP9MDH5MBPbIqV92AaeXatLxBI9gBaebbnrfifHhDYfgasaacH8akY=wiFfYdH8Gipec8Eeeu0xXdbba9frFj0=OqFfea0dXdd9vqai=hGuQ8kuc9pgc9s8qqaq=dirpe0xb9q8qiLsFr0=vr0=vr0dc8meaabaqaciaacaGaaeqabaqabeGadaaakeaafaqabeqacaaabaacbaGae83CamNae8xzauMae8NBa4Mae83CamNae8xAaKMae8hDaqNae8xAaKMae8NDayNae8xAaKMae8hDaqNae8xEaKNae8NoaOdabaacciGae4hYdKNaeiikaGIaem4CamNaeiilaWIaemOCaiNaeiykaKIaeyypa0ZaaSaaaeaadaabdaqaaiabfc6aqjabcIcaOiabdohaZjabcMcaPiabgMIihlabdkhaYbGaay5bSlaawIa7aaqaamaaemaabaGaemOCaihacaGLhWUaayjcSdaaaaaacaWLjaGaaCzcamaabmaabaGaeGOmaidacaGLOaGaayzkaaaaaa@587A@

score:σ(s,r)=|Π(s)∩r||Π(s)∪r|     (3)
 MathType@MTEF@5@5@+=feaafiart1ev1aaatCvAUfKttLearuWrP9MDH5MBPbIqV92AaeXatLxBI9gBaebbnrfifHhDYfgasaacH8akY=wiFfYdH8Gipec8Eeeu0xXdbba9frFj0=OqFfea0dXdd9vqai=hGuQ8kuc9pgc9s8qqaq=dirpe0xb9q8qiLsFr0=vr0=vr0dc8meaabaqaciaacaGaaeqabaqabeGadaaakeaafaqabeqacaaabaacbaGae83CamNae83yamMae83Ba8Mae8NCaiNae8xzauMaeiOoaOdabaacciGae43WdmNaeiikaGIaem4CamNaeiilaWIaemOCaiNaeiykaKIaeyypa0ZaaSaaaeaadaabdaqaaiabfc6aqjabcIcaOiabdohaZjabcMcaPiabgMIihlabdkhaYbGaay5bSlaawIa7aaqaamaaemaabaGaeuiOdaLaeiikaGIaem4CamNaeiykaKIaeyOkIGSaemOCaihacaGLhWUaayjcSdaaaaaacaWLjaGaaCzcamaabmaabaGaeG4mamdacaGLOaGaayzkaaaaaa@5637@

Let *S *be the set of suggested families evaluated and *R *be the set of reference families. For each suggested family *s *and each family *r *we define:

σR(s)=max⁡r∈Rσ(s,r)σS(r)=max⁡s∈Sσ(s,r)     (4)
 MathType@MTEF@5@5@+=feaafiart1ev1aaatCvAUfKttLearuWrP9MDH5MBPbIqV92AaeXatLxBI9gBaebbnrfifHhDYfgasaacH8akY=wiFfYdH8Gipec8Eeeu0xXdbba9frFj0=OqFfea0dXdd9vqai=hGuQ8kuc9pgc9s8qqaq=dirpe0xb9q8qiLsFr0=vr0=vr0dc8meaabaqaciaacaGaaeqabaqabeGadaaakeaafaqabeqacaaabaacciGae83Wdm3aaSbaaSqaaiabdkfasbqabaGccqGGOaakcqWGZbWCcqGGPaqkcqGH9aqpdaWfqaqaaiGbc2gaTjabcggaHjabcIha4bWcbaGaemOCaiNaeyicI4SaemOuaifabeaakiab=n8aZHGaaiab+HcaOGqaciab9nhaZjab9XcaSiab9jhaYHqaaiab8LcaPaqaaiab=n8aZnaaBaaaleaacqqFtbWuaeqaaOGaeiikaGIaemOCaiNaeiykaKIaeyypa0ZaaCbeaeaacyGGTbqBcqGGHbqycqGG4baEaSqaaiabdohaZjabgIGiolabdofatbqabaGccqWFdpWCcqGFOaakcqqFZbWCcqqFSaalcqqFYbGCcqaFPaqkaaGaaCzcaiaaxMaadaqadaqaaiabisda0aGaayjkaiaawMcaaaaa@5E64@

*σ*_*S*_(*r*) measures how well the evaluated system *S *can reconstruct *R*. *σ*_*R*_(*s*) measures how well cluster *s *can be explained by the reference set *R*. *σ*_*R*_(*s*) is the target of the regression function trained in stage 6.

For the evaluation presented in section **Results **we use several other quantities:

φ(s)=φ(s,arg⁡max⁡r∈Rσ(s,r))     (5)
 MathType@MTEF@5@5@+=feaafiart1ev1aaatCvAUfKttLearuWrP9MDH5MBPbIqV92AaeXatLxBI9gBaebbnrfifHhDYfgasaacH8akY=wiFfYdH8Gipec8Eeeu0xXdbba9frFj0=OqFfea0dXdd9vqai=hGuQ8kuc9pgc9s8qqaq=dirpe0xb9q8qiLsFr0=vr0=vr0dc8meaabaqaciaacaGaaeqabaqabeGadaaakeaaiiGacqWFgpGzcqGGOaakcqWGZbWCcqGGPaqkcqGH9aqpcqWFgpGzcqGGOaakcqWGZbWCcqGGSaalcyGGHbqycqGGYbGCcqGGNbWzdaWfqaqaaiGbc2gaTjabcggaHjabcIha4bWcbaGaemOCaiNaeyicI4SaemOuaifabeaakiab=n8aZHGaaiab+HcaOGqaciab9nhaZjab9XcaSiab9jhaYjab+LcaPiabcMcaPiaaxMaacaWLjaWaaeWaaeaacqaI1aqnaiaawIcacaGLPaaaaaa@4FCA@

ψ(s)=ψ(s,argmaxr∈Rσ(s,r))     (6)
 MathType@MTEF@5@5@+=feaafiart1ev1aaatCvAUfKttLearuWrP9MDH5MBPbIqV92AaeXatLxBI9gBaebbnrfifHhDYfgasaacH8akY=wiFfYdH8Gipec8Eeeu0xXdbba9frFj0=OqFfea0dXdd9vqai=hGuQ8kuc9pgc9s8qqaq=dirpe0xb9q8qiLsFr0=vr0=vr0dc8meaabaqaciaacaGaaeqabaqabeGadaaakeaaiiGacWaJa+hYdKhccaGae4hkaGIaem4CamNae4xkaKIae4xpa0Jae8hYdKNae4hkaGccbiGae03CamNae0hlaWccbaGaeWxyaeMaeWNCaiNaeW3zaC2aaCbeaeaacqaFTbqBcqaFHbqycqaF4baEaSqaaiab9jhaYjabgIGiolab9jfasbqabaGccqWFdpWCcqGFOaakcqqFZbWCcqqFSaalcqqFYbGCcqGFPaqkcqqFGaaicqGFPaqkcaWLjaGaaCzcaiab+HcaOiab+zda2iab+LcaPaaa@5196@

φ(r)=φ(arg⁡max⁡s∈Sσ(s,r),r)     (7)
 MathType@MTEF@5@5@+=feaafiart1ev1aaatCvAUfKttLearuWrP9MDH5MBPbIqV92AaeXatLxBI9gBaebbnrfifHhDYfgasaacH8akY=wiFfYdH8Gipec8Eeeu0xXdbba9frFj0=OqFfea0dXdd9vqai=hGuQ8kuc9pgc9s8qqaq=dirpe0xb9q8qiLsFr0=vr0=vr0dc8meaabaqaciaacaGaaeqabaqabeGadaaakeaaiiGacqWFgpGziiaacqGFOaakieGacqqFYbGCcqGFPaqkcqGF9aqpcqWFgpGzcqGGOaakcyGGHbqycqGGYbGCcqGGNbWzdaWfqaqaaiGbc2gaTjabcggaHjabcIha4bWcbaGaem4CamNaeyicI4Saem4uamfabeaakiab=n8aZjabcIcaOiabdohaZjabcYcaSiabdkhaYjabcMcaPiabcYcaSiabdkhaYjabcMcaPiaaxMaacaWLjaGaeiikaGIaeG4naCJaeiykaKcaaa@4FFA@

ψ(r)=ψ(arg⁡max⁡s∈Sσ(s,r),r)     (8)
 MathType@MTEF@5@5@+=feaafiart1ev1aaatCvAUfKttLearuWrP9MDH5MBPbIqV92AaeXatLxBI9gBaebbnrfifHhDYfgasaacH8akY=wiFfYdH8Gipec8Eeeu0xXdbba9frFj0=OqFfea0dXdd9vqai=hGuQ8kuc9pgc9s8qqaq=dirpe0xb9q8qiLsFr0=vr0=vr0dc8meaabaqaciaacaGaaeqabaqabeGadaaakeaaiiGacqWFipqEcqGGOaakcqWGYbGCcqGGPaqkcqGH9aqpcqWFipqEcqGGOaakcyGGHbqycqGGYbGCcqGGNbWzdaWfqaqaaiGbc2gaTjabcggaHjabcIha4bWcbaGaem4CamNaeyicI4Saem4uamfabeaakiab=n8aZjabcIcaOiabdohaZjabcYcaSiabdkhaYjabcMcaPiabcYcaSiabdkhaYjabcMcaPiaaxMaacaWLjaGaeiikaGIaeGioaGJaeiykaKcaaa@5027@

*φ*(*s*) and *ψ*(*s*) are the selectivity and sensitivity of a given suggested family with respect to the best matching reference family. *φ*(*r*) and *ψ*(*r*) are the selectivity and sensitivity of the maximal scoring suggested family with respect to a given reference family.

### Computational resources

The EVEREST process was run on a grid of ~300 machines of different models running MOSIX Linux [30].

## Authors' contributions

EP, ML and NL conceived the project and wrote this manuscript. EP developed and programmed the process and performed the experiments. AH has recoded the final version of the programs. ML and NL guided and directed the research.

## Figures and Tables

**Table 1:** Performance of EVEREST release 1, ProtoNet and ADDA with respect to Pfam

Thresholds		Accuracy			Hetero Family Coverage				
Selectivity	Sensitivity	EVEREST	ProtoNet	ADDA	EVEREST			ProtoNet	ADDA
					All	Train	Test		
50%	50%	62%	32%	25%	71%	71%	72%	41%	53%
50%	75%	52%	27%	22%	66%	65%	66%	35%	45%
50%	87.5%	42%	24%	20%	59%	58%	59%	29%	39%
50%	93.75%	35%	23%	19%	52%	52%	52%	25%	35%
75%	50%	51%	27%	23%	65%	65%	65%	24%	42%
75%	75%	43%	23%	20%	60%	59%	60%	19%	34%
75%	87.5%	35%	21%	18%	52%	52%	53%	14%	29%
75%	93.75%	29%	19%	18%	46%	46%	46%	11%	26%
87.5%	50%	43%	26%	23%	60%	60%	59%	19%	38%
87.5%	75%	36%	21%	19%	55%	56%	54%	14%	31%
87.5%	87.5%	30%	19%	18%	48%	49%	47%	10%	26%
87.5%	93.75%	24%	18%	17%	42%	43%	41%	07%	23%
93.75%	50%	37%	25%	22%	55%	56%	54%	16%	34%
93.75%	75%	31%	21%	19%	50%	51%	50%	11%	27%
93.75%	87.5%	26%	19%	18%	44%	44%	44%	07%	23%
93.75%	93.75%	21%	18%	17%	38%	39%	37%	05%	21%

## References

[B1] Portugaly E, Savenok A, Linial N, Linial M (2006). EVEREST. http://www.everest.cs.huji.ac.il/.

[B2] Wu CH, Apweiler R, Bairoch A, Natale DA, Barker WC, Boeckmann B, Ferro S, E G, Huang H, Lopez R, Magrane M, Martin MJ, Mazumder R, O'Donovan C, Redaschi N, Suzek B (2006). The Universal Protein Resource (UniProt): an expanding universe of protein information. Nucleic Acids Res.

[B3] Altschul SF, Madden TL, Schaffer AA, Zhang J, Zhang Z, Miller W, Lipman DJ (1997). Gapped BLAST and PSI-BLAST: a new generation of protein database search programs. Nucleic Acids Res.

[B4] Berman HM, Westbrook J, Feng Z, Gilliland G, Bhat TN, Weissig H, Shindyalov IN, Bourne PE (2000). The Protein Data Bank. Nucleic Acids Res.

[B5] Inbar Y, Benyamini H, Nussinov R, Wolfson HJ (2003). Protein structure prediction via combinatorial assembly of sub-structural units. Bioinformatics.

[B6] Liu J, Rost B (2003). Domains, motifs and clusters in the protein universe. Curr Opin Chem Biol.

[B7] Bateman A, Birney E, Cerruti L, Durbin R, Etwiller L, Eddy SR, Griffiths-Jones S, Howe KL, Marshall M, Sonnhammer EL (2002). The Pfam protein families database. Nucleic Acids Res.

[B8] Schultz J, Copley RR, Doerks T, Ponting CP, Bork P (2000). SMART: a web-based tool for the study of genetically mobile domains. Nucleic Acids Res.

[B9] Hubbard TJ, Ailey B, Brenner SE, Murzin AG, Chothia C (1999). SCOP: a Structural Classification of Proteins database. Nucleic Acids Res.

[B10] Gracy J, Argos P (1998). DOMO: a new database of aligned protein domains. Trends Biochem Sci.

[B11] Servant F, Bru C, Carrere S, Courcelle E, Gouzy J, Peyruc D, Kahn D (2002). ProDom: automated clustering of homologous domains. Brief Bioinform.

[B12] Heger A, Holm L (2003). Exhaustive enumeration of protein domain families. J Mol Biol.

[B13] Gracy J, Argos P (1998). Automated protein sequence database classification. I. Integration of compositional similarity search, local similarity search, and multiple sequence alignment. Bioinformatics.

[B14] Nagarajan N, Yona G (2004). Automatic prediction of domains from sequence information using hybrid learning system. Bioinformatics.

[B15] Liu J, Rost B (2004). CHOP: parsing proteins into structural domains. Nucleic Acids Res.

[B16] Sasson O, Vaaknin A, Fleischer H, Portugaly E, Bilu Y, Linial N, Linial M (2003). ProtoNet: hierarchical classification of the protein space. Nucleic Acids Res.

[B17] Dekel O, Shalev-Shwartz S, Singer Y (2003). Smooth Epsilon- Insensitive Regression by Loss Symmetrization. Proceedings of the Sixteenth Annual Conference on Computational Learning Theory.

[B18] Eddy SR (2001). HMMER: Profile hidden Markov models for biological sequence analysis. http://hmmer.wustl.edu/.

[B19] Park J, Karplus K, Barrett C, Hughey R, Haussler D, Hubbard T, Chothia C (1998). Sequence comparisons using multiple sequences detect three times as many remote homologues as pairwise methods. J Mol Biol.

[B20] Sasson O, Linial N, Linial M (2002). The metric space of proteins-comparative study of clustering algorithms. Bioinformatics.

[B21] Shachar O, Linial M (2004). A robust method to detect structural and functional remote homologues. Proteins: Structure, Function, and Bioinformatics.

[B22] Kaplan N, Friedlich M, Fromer M, Linial M (2004). A functional hierarchical organization of the protein sequence space. BMC Bioinformatics.

[B23] Bairoch A (2000). The ENZYME database in 2000. Nucleic Acids Res.

[B24] Mulder NJ, Apweiler R, Attwood TK, Bairoch A, Bateman A, Binns D, Biswas M, Bradley P, Bork P, Bucher P, Copley R, Courcelle E, Durbin R, Falquet L, Fleischmann W, Gouzy J, Griffith-Jones S, Haft D, Hermjakob H, Hulo N, Kahn D, Kanapin A, Krestyaninova M, Lopez R, Letunic I, Orchard S, Pagni M, Peyruc D, Ponting CP, Servant F, Sigrist CJ (2002). InterPro: an integrated documentation resource for protein families, domains and functional sites. Brief Bioinform.

[B25] Chandonia JM, Walker NS, Lo Conte L, Koehl P, Levitt M, Brenner SE (2002). ASTRAL compendium enhancements. Nucleic Acids Res.

[B26] Boeckmann B, Bairoch A, Apweiler R, Blatter MC, Estreicher A, Gasteiger E, Martin MJ, Michoud K, O'Donovan C, Phan I, Pilbout S, Schneider M (2003). The SWISS-PROT protein knowledgebase and its supplement TrEMBL in 2003. Nucleic Acids Res.

[B27] Smith TF, Waterman MS (1981). Identification of common molecular subsequences. J Mol Biol.

[B28] Thompson JD, Higgins DG, J GT (1994). CLUSTAL W: improving the sensitivity of progressive-multiple sequence alignment through sequence weighting, position-specific gap penalties and weight matrix choice. Nucleic Acids Res.

[B29] Portugaly E, Ninio M (2004). HMMERHEAD – Accelerating HMM Searches On Large Databases. Currents in Computational Molecular Biology – Poster Abstracts from RECOMB.

[B30] Barak A, Shiloh A, Amar L (2005). An Organizational Grid of Federated MOSIX Clusters. CCGrid05 – Proc 5th IEEE International Symposium on Cluster Computing and the Grid.

